# Changes in hamstring strength after anterior cruciate ligament reconstruction with hamstring autograft and posterior cruciate ligament reconstruction with tibialis allograft

**DOI:** 10.1186/s43019-020-00047-2

**Published:** 2020-06-05

**Authors:** O-Sung Lee, Yong Seuk Lee

**Affiliations:** 1 Department of Orthopaedic Surgery, Mediplex Sejong Hospital, Incheon, South Korea; 2 Department of Orthopaedic Surgery, Seoul National University Bundang Hospital, Seongnam-si, South Korea

**Keywords:** Anterior cruciate ligament, Posterior cruciate ligament, Reconstruction, Hamstring autograft, Tibialis allograft, Hamstring strength

## Abstract

**Aim:**

The aim of this study was to evaluate the changes in hamstring strength both after anterior cruciate ligament reconstruction (ACLR) with hamstring autograft followed by early rehabilitation and posterior cruciate ligament reconstruction (PCLR) with tibialis allograft followed by delayed rehabilitation.

**Methods:**

Isokinetic strengths of the quadriceps and hamstring muscles and endurances were compared between a group of 20 patients undergoing PCLR using a tibialis anterior allograft and a 1:2 matched control group of 40 patients undergoing ACLR using a hamstring autograft at 2 years after the operations. Clinical results were also compared using stability tests and the Lysholm and the International Knee Documentation Committee scores.

**Results:**

At 2 years after the operations, the torque deficit of the hamstring muscle in the involved leg compared to the uninvolved leg at both 60°/s and 120°/s was greater in the PCLR group than in the ACLR group (60°/s, 21.8 ± 14.0% versus 1.9 ± 23.9%, *P* = 0.0171; 120°/s, 15.3 ± 13.7% versus −0.7 ± 17.4%, *p* = 0.012, respectively). The peak torque of the hamstring muscle at 120°/s was significantly lower in the involved leg than in the uninvolved leg only in the PCLR group (71.3 ± 31.9 N∙m versus 81.9 ± 27.8 N∙m, *P* = 0.005). There was no significant difference in the clinical results between the groups except for a side-to-side difference in the tibial translation on Telos stress radiographs.

**Conclusion:**

The strength of the hamstring of the PCLR leg with allograft was significantly weaker than that of the unoperated leg after 2 years, whereas that of the ACLR leg with hamstring autograft maintained a similar level of strength compared to that of the uninvolved leg.

**Level of evidence:**

Level III, case–control study.

## Introduction

In terms of restoration of knee function after anterior cruciate ligament reconstruction (ACLR) and posterior cruciate ligament reconstruction (PCLR), an improved understanding of the rehabilitation and recovery pattern of the muscles may be required [1–3]. Proper rehabilitation after ACLR results in good stability of the knee joint as well as the recovery of muscle power and sufficient range of motion (ROM) [4–8]. Regardless of the types of grafts used, excellent results regarding functional outcome and activity level have been reported with no differences between them [2, 9–13]. However, concerns over each graft type still exist, and certain advantages and disadvantages according to each graft type have been suggested [14–16]. In addition, ACLR using a hamstring autograft can give rise to a greater loss of knee flexor strength after surgery [14, 15].

Outcomes after PCLR have been reported to be inferior to those after ACLR [3, 17–19]. Recently, excellent functional outcomes after PCLR have been reported, with patients showing a return to their pre-injury level of activity because of the improved understanding of rehabilitation as well as advances in surgical techniques [17, 20–24]. However, a more conservative rehabilitation is preferred after PCLR than after ACLR [18, 20, 21]. Patients should avoid active flexion exercises after PCLR because these can result in posterior translation of the tibia and interfere with the healing process of related tissues [3, 19]. Although the hamstring tendon could be saved during PCLR if other graft materials, such as tibialis anterior (TA) allografts, are used, there is a still concern regarding hamstring muscle weakness owing to inhibition of hamstring muscle exercises during the early postoperative period. However, information on the isokinetic changes in muscle strength and endurance in patients after PCLR is still lacking. In addition, the comparison of muscle strength and endurance after ACLR and PCLR has not been reported.

The purpose of this study was to evaluate the changes in hamstring strength after ACLR with hamstring autograft followed by early rehabilitation versus PCLR with tibialis allograft followed by delayed rehabilitation. We hypothesized that the strength of the hamstring muscles in both groups would be lower in the affected leg than in the unaffected leg.

## Materials and methods

### Patient selection

This study compared the data that were obtained from patients who underwent ACLR with hamstring autograft and PCLR with TA allograft between 2014 and 2015. The ACLR and PCLR groups underwent a primary unilateral ligament reconstruction due to acute anterior cruciate ligament (ACL) or posterior cruciate ligament (PCL) injury without other major ligament or osseous surgical procedures. All tears of the ligament were observed by magnetic resonance imaging, and the diagnoses were confirmed using intraoperative arthroscopy. All ACLRs were performed using the trans-septal technique and hamstring muscle autograft and all PCLRs were performed using the trans-tibial technique and TA allograft [25, 26]. This study included only patients within an interval of 6 weeks from trauma to surgery because the preoperative muscle condition in the chronic injury would vary with the time interval. Patients with concomitant meniscus tears and ligament injuries, which required other specific rehabilitation protocols other than the routine protocol, were excluded.

Seventy-nine patients treated via ACLR and 28 patients treated via PCLR met the inclusion criteria. A retrospective 1:2 matched-pair comparison was conducted. For each patient in the PCLR group, two patients were selected from the ACLR group, with the matching criteria of age (±1 year), sex and body mass index (±3 kg/m^2^). When there was no or only one patient in the ACLR group who matched the selection criteria for a patient in the PCLR group, all the patients were excluded. Finally, a total of 40 patients who underwent ACLR and 20 patients who underwent PCLR and who completed the isokinetic testing and clinical evaluation preoperatively and 2 years postoperatively were successfully included in this study. The demographic data of the two groups are shown in Table 1. There was no significant difference between the two groups in terms of age, height, weight, body mass index, sex, dominant side, incidence of meniscal repair and interval from trauma to surgery. This study obtained the approval of our institutional review board, and none of the isokinetic results of this series have been used in other studies.

**Table 1:** Patient demographics

	ACLR group	PCLR group	*P* value
Patients (knees), *n*	40	20	
Age (years)	30.7 ± 10.4	31.4 ± 11.2	0.248*
Height (cm)	172.8 ± 5.4	173.8 ± 6.2	0.607*
Weight (kg)	75.4 ± 11.7	76.5 ± 10.2	0.286*
BMI (kg/m^2^)	25.3 ± 3.4	25.4 ± 3.6	0.787*
Sex (male/female), *n*	38/2	19/1	1.000**
Side (dominant/non-dominant), *n*	24/16	14/6	0.573**
Meniscal repair, *n*	5 (12.5%)	2 (10%)	1.000**
Interval from trauma to surgery (weeks)	4.0 ± 1.3	4.2 ± 1.7	0.487*

Values are presented as mean ± standard deviation unless otherwise indicated

The statistical significance was set at *P* < 0.05

*ACLR* anterior cruciate ligament reconstruction, *BMI* body mass index, *PCLR* posterior cruciate ligament reconstruction

*Derived with Student’s *t* test

**Derived with Pearson chi-square test

### Isokinetic test

The isokinetic strength of the involved and uninvolved legs was measured using the BTE PrimusRS™ (Baltimore Therapeutic Equipment, Maryland and Colorado, USA) preoperatively and at 2 years postoperatively. The patients were seated in an upright position with a 90° hip flexion on the testing device. After sitting, the chest, pelvis and thigh were immobilized using straps. After the uninvolved leg was tested, the involved leg was then tested. The ROM of the knee joint was set from 0° to 70°. After warm-up repetitions, the measurements were repeated five times at an angular velocity of 60°/s and 25 times at 120°/s. Peak torque was defined as the maximum value during the repetitions (N∙m) of flexion and extension. The extension and flexion peak torques of the involved leg were compared with those of the uninvolved leg, and the percentage of the torque deficit of each muscle in the involved leg compared to the uninvolved leg was also recorded. The hamstring to quadriceps ratio was calculated as the measurement of knee muscle balance. To determine muscular endurance, the total work was recorded as the work produced by the repetitions of consecutive extension and flexion of the knee joint at each angular velocity. All values measured during extension represented the quadriceps muscle strength, and those during flexion represented the hamstring muscle strength.

### Postoperative rehabilitation

All patients in the two groups followed the home-based and standardized rehabilitation protocol according to the kind of reconstruction surgery they received. Regular follow-ups were performed at 2 weeks, 6 weeks, 3 months, 6 months, 1 year, and every year thereafter to provide adequate rehabilitation for each period. The ACLR group was permitted full weight bearing using a brace immediately after surgery. The goal for the patients was to gain 120° of ROM 6 weeks after surgery. Closed kinetic chain exercises were started 6 weeks after surgery, and open kinetic chain exercises were started 12 weeks after surgery. A perturbation training program was started 6 weeks after surgery. Running was allowed at 3 months, with a return to sports activities 6 months after surgery. The PCLR group was permitted partial weight bearing using a brace immediately after surgery. Passive flexion exercises of the knee joint were permitted immediately after surgery. The ROM gradually increased to 90° at 6 weeks. Hamstring muscle strengthening exercises were started at 12 weeks. Table 2 shows the routine rehabilitation protocols of our hospital for ACLR and PCLR in detail.

**Table 2:** Rehabilitation program for ACLR and PCLR

Postoperative period		ACLR	PCLR
Early postoperative phase (0–6 weeks)	Joint mobility	0–90° ROM exercise until 2 weeks 120° ROM increase until 6 weeks Manual patellar mobilization	Immediate immobilization in full extension with posterior pad Gradual increase to ROM 90° until 6 weeks Manual patellar mobilization
	Weight bearing and brace	Immediate full weight bearing with brace	Partial weight bearing with 0° locked brace
	Exercise	Active quadriceps (straight leg raise, isometric quadriceps sets) Active hamstring exercise (hamstring sets, standing hamstring curls at 2 weeks) Ankle pump	Supine passive ROM with both hands support Prone passive flexion exercise Calf raise and isometric quadriceps sets
	Functional goal	Normal gait pattern with single clutch and unlocked brace at 2 weeks Normal gait pattern without assistance and brace at 6 weeks	Early protected ROM Caution against posterior tibial translation by gravity, muscle action
Intermediate postoperative phase (6–12 weeks)	Joint mobility	Achieve more than 120° ROM	90–120° ROM exercise until 12 weeks
	Weight bearing and brace	Brace off and start full weight bearing at postoperative 6 weeks	Start full weight bearing with brace at 6 weeks
	Exercise	CKC exercise (squat 0–60°, lunge 0–60°, leg press with gradual progressive resistance), stationary bike, stairs (concentric and eccentric)	Continue ROM exercise Quad sets, single-leg squat, calf raise
	Functional goal	Single-leg squat to 60°, equal quad girth	Normal gait pattern without assistance and brace at 12 weeks Increase ROM
Return to activity phase (12 weeks to 6 months)	Joint mobility	Full ROM	Achieve more than 120° ROM exercise
	Weight bearing & brace		Brace off and start full weight bearing at 12 weeks
	Exercise	OKC exercise Shuttle running, jumping rope, light running, aqua jogging	Hamstring strengthening exercise Start CKC exercise (mini-squat 0–45°, wall slides, leg press 0–45°) Progress CKC exercise Straight line running, swimming (no frog kick), jogging in pool
	Criteria to progress to next phase	Single-leg full squat Single leg stance at least 60 s Good landing form with bilateral vertical and horizontal jumping	Full and pain-free ROM, normal gait, good to normal quadriceps strength, no patellofemoral complaints
Return to sports activity (after 6 months)	Exercise	Progressive running program, hop testing and training, progressive plyometrics, competitive sports, progress to sports-specific drills	Light sports, progress jogging and running Competitive sports after 9 months

*ACLR* anterior cruciate ligament reconstruction, *CKC* closed kinetic chain, *OKC* open kinetic chain, *PCLR* posterior cruciate ligament reconstruction, *ROM* range of motion

### Clinical evaluation

The manual laxity was evaluated based on the anterior drawer test and pivot-shift test for the ACLR group, and the posterior drawer test for the PCLR group preoperatively and at every follow-up. As an indicator of knee stability, the side-to-side difference in the anterior translation on the Telos stress radiograph was used for the ACLR group and the difference in the posterior translation was used for the PCLR group. Flexion contracture and active maximal flexion were measured in the supine position using a goniometer. The clinical status was evaluated 1 day before surgery and every year after surgery using the Lysholm score and subjective and objective International Knee Documentation Committee (IKDC) scores.

### Statistical analysis

All statistical analyses were performed using SPSS version 22.0 (IBM Corp., Armonk, NY, USA). Data description was based on means and standard deviations for continuous variables. The differences in continuous variables were analyzed using the Student’s *t* test or the Mann–Whitney test according to the appropriate normality tests. The differences in other categorical variables were analyzed with Pearson’s chi-square test or Fisher exact test or linear-by-linear association. Statistical significance was set at *P* < 0.05. A post-hoc power analysis was performed to assess the validity of the number of patients required in each group according to each parameter (α = 0.05, power = 80%).

## Results

All isokinetic data of the patients who completed a 2-year follow-up are shown in Table 3. There were no statistically significant differences between groups in any preoperative measurements.

**Table 3:** Isokinetic strength and endurance in quadriceps and hamstring

			Preoperative			Postoperative 2 year		
			ACLR	PCLR	*P* value	ACLR	PCLR	*P* value
60°/s	Quadriceps peak torque, N∙m	Involved	66.5 ± 35.5	75.2 ± 45.1	0.430	100.1 ± 33.7	104.6 ± 44.1	0.748
		Uninvolved	108.2 ± 35.6	100.0 ± 39.8	0.428	114.3 ± 33.8	121.7 ± 52.1	0.625
	Hamstring peak torque, N∙m	Involved	52.7 ± 27.0	49.5 ± 24.4	0.659	73.7 ± 22.1	71.3 ± 31.9	0.802
		Uninvolved	70.4 ± 25.4	66.7 ± 28.5	0.617	78.4 ± 26.6	81.9 ± 27.8	0.727
	Torque deficit in the involved leg compared to uninvolved leg (%)	Quadriceps	36.4 ± 34.6	25.5 ± 31.8	0.253	9.8 ± 24.5	19.4 ± 29.1	0.328
		Hamstring	21.9 ± 37.4	25.4 ± 27.5	0.723	1.9 ± 23.9	21.8 ± 14.0	**0.017**
	Hamstring to quadriceps ratio	Involved	0.82 ± 0.20	0.73 ± 0.28	0.157	0.79 ± 0.31	0.80 ± 0.19	0.982
		Uninvolved	0.66 ± 0.13	0.70 ± 0.19	0.384	0.70 ± 0.16	0.80 ± 0.32	0.227
	Work, J	Involved	473.8 ± 278.6	433.7 ± 269.7	0.604	695.0 ± 237.0	578.2 ± 350.7	0265
		Uninvolved	756.7 ± 308.1	709.4 ± 264.9	0.566	819.0 ± 225.8	812.5 ± 325.1	0.947
120°/s	Quadriceps peak torque, N∙m	Involved	56.8 ± 2885	61.4 ± 36.7	0.613	77.0 ± 28.2	73.4 ± 35.2	0.752
		Uninvolved	79.5 ± 32.0	72.5 ± 33.0	0.443	83.1 ± 25.5	90.1 ± 32.2	0.501
	Hamstring peak torque, N∙m	Involved	52.9 ± 24.8	48.6 ± 29.7	0.562	61.8 ± 19.2	62.7 ± 26.0	0.915
		Uninvolved	59.8 ± 23.7	58.1 ± 31.1	0.819	62.1 ± 19.3	73.9 ± 29.4	0.174
	Torque deficit in the involved leg compared to uninvolved leg (%)	Quadriceps	28.3 ± 21.4	9.9 ± 50.1	0.131	6.8 ± 22.5	17.1 ± 29.1	0.269
		Hamstring	12.5 ± 22.1	11.6 ± 41.1	0.917	−0.7 ± 17.4	15.3 ± 13.7	**0.012**
	Hamstring to quadriceps ratio	Involved	1.02 ± 0.51	0.83 ± 0.27	0.122	0.89 ± 0.49	0.91 ± 0.21	0.926
		Uninvolved	0.82 ± 0.47	0.80 ± 0.13	0.810	0.76 ± 0.13	0.84 ± 0.23	0.189
	Work, J	Involved	1930.7 ± 1163.6	1657.7 ± 1088.7	0.393	2724.4 ± 994.3	2422.5 ± 1189.2	0.447
		Uninvolved	2994.6 ± 1113.7	2443.7 ± 1167.0	0.087	2902.1 ± 1083.0	3108.5 ± 1174.5	0.619

Values are presented as mean ± standard deviation derived with Student’s *t* test

The statistical significance was set at *P* < 0.05; significant results are shown in bold type

*ACLR* anterior cruciate ligament reconstruction, *PCLR* posterior cruciate ligament reconstruction

In terms of the values measured postoperatively at 2 years, the torque deficit of the hamstring muscle in the involved leg compared to the uninvolved leg at both 60°/s and 120°/s was greater in the PCLR group than in the ACLR group (60°/s, 21.8 ± 14.0% versus 1.9 ± 23.9%, *P* = 0.0171; 120°/s, 15.3 ± 13.7% versus −0.7 ± 17.4%, *P* = 0.012, respectively), although there was no statistically significant difference in the absolute value of the hamstring peak torque between the groups. Additionally, there were no statistically significant differences between the groups regarding the peak torque of the quadriceps, torque deficit of the quadriceps muscle in the involved leg compared to the uninvolved leg, hamstring to quadriceps ratio or total work of both legs.

**Fig. 1:**
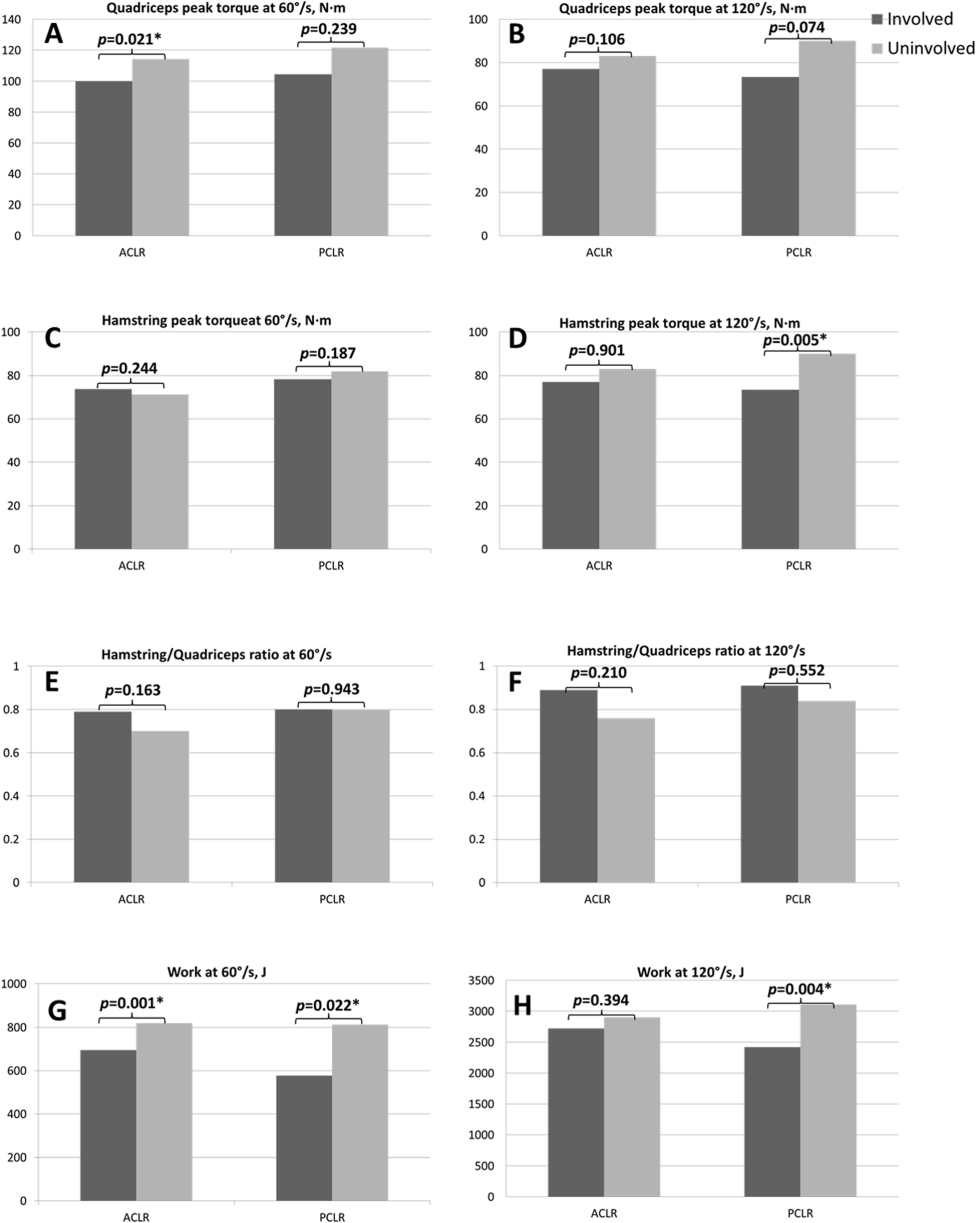
Comparison of the isokinetic evaluations between the involved and uninvolved legs in the anterior cruciate ligament reconstruction (ACLR) and posterior cruciate ligament reconstruction (PCLR) groups. **a** Quadriceps peak torque at 60°/s; **b** quadriceps peak torque at 120°/s; **c** hamstring peak torque at 60°/s; **d** hamstring peak torque at 120°/s; **e** hamstring to quadriceps ratio at 60°/s; **f** hamstring to quadriceps ratio at 120°/s; **g** work at 60°/s; **h** work at 120°/s. *Significant difference at *P* < 0.05

In addition, the involved and uninvolved legs in each group were compared. Peak torque of the hamstring muscle at 120°/s was significantly lower in the involved leg than in the uninvolved leg only in the PCLR group (71.3 ± 31.9 N∙m versus 81.9 ± 27.8 N∙m, *P* = 0.005). Total work at 60°/s was lower in the involved leg than in the uninvolved leg in both ACLR and PCLR groups (ACLR, 695.0 ± 237.0 versus 819.0 ± 225.8, *P* = 0.001; PCLR, 578.2 ± 350.7 versus 812.5 ± 325.1, *P* = 0.022, respectively), and at 120°/s was significantly lower in the involved leg than in the uninvolved leg only in the PCLR group (2422.5 ± 1189.2 versus 3108.5 ± 1174.5, *P* = 0.004) (Table 3 and Fig. 1). Additionally, peak torque of the quadriceps muscle at 60°/s was significantly lower in the involved leg than in the uninvolved leg in the ACLR group (100.1 ± 33.7 N∙m versus 114.3 ± 33.8 N∙m, *P* = 0.021).

In terms of the clinical results, there were no statistically significant differences between the two groups. Improvements in manual laxity tests were observed in both groups. There was no statistically significant difference in the postoperative values of ROM, Lysholm scores (ACLR versus PCLR, 86.6 ± 14.4 versus 86.8 ± 8.3, *P* = 0.971) or subjective IKDC scores (ACLR versus PCLR, 90.4 ± 16.4 versus 84.3 ± 11.3, *P* = 0.548). The objective IKDC scores also showed a similar result in the two groups (*P* = 0.104). Postoperative side-to-side differences in anterior tibial translation in the ACLR group was greater than that of posterior tibial translation in the PCLR group (2.1 ± 1.7 mm versus 4.1 ± 2.6 mm, *P* = 0.045). However, the difference in the tibial translation between the groups has been widely accepted as a trait associated with each ACLR and PCLR surgery (Table 4).

**Table 4:** Comparison of clinical outcomes between groups

	ACLR group	PCLR group	*P* value
Anterior drawer test (grade 0/1/2/3)			
Preoperative	0/15/22/3	N/A	
Postoperative	27/13/1/0	N/A	
Pivot-shift test (grade 0/1/2/3)			
Preoperative	1/20/16/3	N/A	
Postoperative	34/6/0/0	N/A	
Posterior drawer test (grade 0/1/2/3)			
Preoperative	N/A	0/8/11/1	
Postoperative	N/A	5/11/4/0	
Range of motion (°)			
Flexion contracture	2.4 ± 2.1	2.1 ± 1.8	0.472*
Maximal flexion	138.4 ± 8.1	137.2 ± 8.1	0.378*
Side-to-side difference of anterior and posterior tibial translation (mm)			
Preoperative	7.4 ± 2.7	12.8 ± 3.7	0.027*
Postoperative	2.1 ± 1.7	4.1 ± 2.6	0.045*
Lysholm score			
Preoperative	58.3 ± 22.4	50.3 ± 16.4	0.162*
Postoperative	86.6 ± 14.4	86.8 ± 8.3	0.971*
IKDC subjective score			
Preoperative	55.4 ± 15.9	44.8 ± 13.4	0.013*
Postoperative	88.1 ± 12.9	86.1 ± 9.7	0.548*
IKDC objective score			
Preoperative (A/B/C/D)	0/0/22/18	0/0/4/16	0.013**
Postoperative (A/B/C/D)	27/12/1/0	9/9/2/0	0.104**

Values are presented as mean ± standard deviation

The statistical significance was set at *P* < 0.05

*ACLR* anterior cruciate ligament reconstruction, *IKDC* International Knee Documentation Committee, *N/A* not applicable, *PCLR* posterior cruciate ligament reconstruction

*Derived with Student’s *t* test

**Derived by linear-by-linear association

A post-hoc power analysis on the hamstring peak torque at 60°/s and 120°/s showed that a total sample size of 2054 and 10,124 specimens would be needed to achieve 80% power, respectively. The statistical power of the hamstring peak torque at 60°/s and 120°/s was 8.7% and 3.9%, respectively. However, a post-hoc power analysis on the torque deficit of the hamstring muscle in the involved leg compared to the uninvolved leg showed that a total sample size of 17 specimens would be needed at both 60°/s and 120°/s. The statistical power of the torque deficit of the hamstring muscle in the involved leg compared to the uninvolved leg at 60°/s and 120°/s was 81.9% and 82.3%, respectively.

## Discussion

The principal finding of this study was that the peak torque of the hamstring in the leg undergoing PCLR was significantly weaker than that in the unoperated leg after 2 years, whereas that in the leg undergoing ACLR maintained a similar level of strength compared to the uninvolved leg, although the hamstring tendon was harvested only in the ACLR in this study. Additionally, despite the decreased strength of the hamstring muscle in the operated leg compared with the unoperated leg after PCLR, the clinical results after PCLR showed no significant difference compared to that after ACLR at 2 years.

Previous studies have shown decreased muscle strength after ACLR with a hamstring autograft. Keays et al. [27] reported that the recovery of hamstring muscle strength was slower than that of quadriceps muscle strength with a 6-month follow-up after ACLR using a hamstring autograft. Lee et al. [2] reported that the knee flexor strength recovered to 80% compared with the strength of the uninjured leg 1 year after ACLR using an autologous hamstring tendon. A study of 73 patients revealed that a more prominently decreased flexor power still exists at least 2 years after hamstring muscle-harvested ACLR than after allografting [14]. To prevent weakness of the muscles, accelerated rehabilitation, including isokinetic flexor strengthening, has been recommended for patients after ACLR regardless of the type of graft used [8, 10, 12, 28, 29]. A recent systematic review of randomized controlled trials with deficient or reconstructed ACLs reported that the optimal time for the initiation of open kinetic chain exercises is at least 6 weeks post-reconstruction or postinjury [5].

In terms of PCLR, a more conservative rehabilitation has been traditionally used [3, 19–21, 30–32]. Recently, various rehabilitation protocols after PCLR have been suggested for muscle strengthening [23, 31–33]. The starting point of closed chain kinetic exercises varied from the immediate postoperative period to 12 weeks [17]. In terms of flexor strengthening, active hamstring muscle exercises are usually delayed for ≥12 weeks after PCLR, whereas quadriceps muscle exercises are encouraged because of the agonistic function of the PCL [17, 22, 30]. However, some studies suggested that active hamstring muscle exercises delayed for 6–8 weeks of accelerated rehabilitation do not indicate a rapid increment of ROM [17, 19]. Active flexion exercises of 0° to 30° of flexion in which the hamstring muscles cannot produce a posterior shear force were permitted [21, 22].

For early rehabilitation after PCLR, co-strengthening by calf raising, short arc leg press, and mini-squatting exercises could be performed [17, 19]. Despite these efforts to strengthen the extensor and flexor muscles concomitantly, a greater decrement in flexor power would be inevitable after PCLR compared to ACLR owing to the longer hamstring muscle inhibition. However, there is a lack of objective data on isokinetic testing observed after PCLR, and data compared with that of ACLR are even more scarce. A recent study showed no significant differences in preoperative strength and endurance of the hamstring muscles between untreated patients with ACL and PCL tears [1]. However, such a study did not compare the postoperative result of isokinetic testing between the two groups.

In our study, the deficit in the hamstring muscle of the involved leg compared with the uninvolved leg in the PCLR group persisted at 2 years, although there was no statistical difference in the absolute value of the hamstring peak torque between the groups. This finding may be because of the delayed rehabilitation after PCLR. Although we emphasized co-strengthening exercises and performed earlier rehabilitation after PCLR than the traditional PCLR rehabilitation, the expected hamstring muscle weakness persisted compared to the healthy leg. Therefore, further studies should be conducted on early PCLR rehabilitation that can strengthen the hamstring muscles without affecting the stability and function after PCLR. Additionally, we managed all patients with a home-based rehabilitation program in this study. Several studies reported that a home-based rehabilitation program is successful for the return of knee ROM and strength after ACLR [5, 34]. However, there is a lack of comparative study between home-based and physical therapy-supervised rehabilitation after PCLR. Therefore, further studies are necessary to determine whether a home-based rehabilitation is as effective after PCLR as after ACLR.

### Limitations

This study has some limitations. First, this was a retrospective study with a relatively small sample size. However, we matched two groups according to our strict matching criteria and there were no significant differences in the demographic data between the groups. Second, the ROM of the isokinetic testing in this study was set only from 0° to 70°. Therefore, we could not confirm the effect of ACLR and PCLR on deep flexion. Third, the total work was not measured separately for the flexion and extension, and so it was not possible to compare the endurances of the hamstring and quadriceps muscles. Finally, direct comparison between early and delayed rehabilitation protocol within each ACLR and PCLR group is required to determine the exact reason for hamstring deficit only after PCLR.

## Conclusions

The strength of the hamstring in the leg undergoing PCLR with allograft was significantly less than that of the unoperated leg after 2 years, whereas the leg undergoing ACLR with hamstring autograft maintained a similar level of strength compared to that of the uninvolved leg.

## Data Availability

All data generated or analyzed during this study are included in this published article.

## Abbreviations

ACL: Anterior cruciate ligament
ACLR: Anterior cruciate ligament reconstruction
IKDC: International Knee Documentation Committee
PCL: Posterior cruciate ligament
PCLR: Posterior cruciate ligament reconstruction
ROM: Range of motion
TA: Tibialis anterior
